# Ex Vivo Pilot Study of Cardiac Magnetic Resonance Velocity Mapping for Quantification of Aortic Regurgitation in a Porcine Model in the Presence of a Transcatheter Heart Valve

**DOI:** 10.1007/s12265-019-09878-1

**Published:** 2019-03-15

**Authors:** Nynke H. M. Kooistra, Freek Nijhoff, Masieh Abawi, Pierfrancesco Agostoni, Daniël M. Araya Roos, Sjoerd van Tuijl, Niels Blanken, Michiel Voskuil, Pieter A. F. M. Doevendans, Pieter R. Stella, Tim Leiner

**Affiliations:** 1 Department of Cardiology, University Medical Center Utrecht, Utrecht, Heidelberglaan 100, 3508 GA Utrecht, The Netherlands; 2 Department of Cardiology, Hartcentrum ZNA Middelheim, Antwerp, Belgium; 3 Department of Radiology, University Medical Center Utrecht, Utrecht, The Netherlands; 4 LifeTec Group, Eindhoven, The Netherlands

**Keywords:** Transcatheter heart valve, Paravalvular aortic regurgitation, Quantification, Cardiac magnetic resonance velocity mapping

## Abstract

Accuracy of aortic regurgitation (AR) quantification by magnetic resonance (MR) imaging in the presence of a transcatheter heart valve (THV) remains to be established. We evaluated the accuracy of cardiac MR velocity mapping for quantification of antegrade flow (AF) and retrograde flow (RF) across a THV and the optimal slice position to use in cardiac MR imaging. In a systematic and fully controlled laboratory ex vivo setting, two THVs (Edwards SAPIEN XT, Medtronic CoreValve) were tested in a porcine model (*n* = 1) under steady flow conditions. Results showed a high level of accuracy and precision. For both THVs, AF was best measured at left ventricular outflow tract level, and RF at ascending aorta level. At these levels, MR had an excellent repeatability (ICC > 0.99), with a tendency to overestimate (4.6 ± 2.4% to 9.4 ± 7.0%). Quantification of AR by MR velocity mapping in the presence of a THV was accurate, precise, and repeatable in this pilot study, when corrected for the systematic error and when the best MR slice position was used. Confirmation of these results in future clinical studies would be a step forward in increasing the accuracy of the assessment of paravalvular AR severity.

## Introduction

Transcatheter aortic valve implantation (TAVI) is a well-accepted alternative treatment for patients with symptomatic, severe native aortic valve stenosis who are at high risk for surgical aortic valve replacement (SAVR) [1]. TAVI may even outperform SAVR, as demonstrated by lower mortality rates in high-risk patients [2, 3].

However, aortic regurgitation (AR) after TAVI remains an important challenge [4, 5]. With respect to the incidence and severity of postoperative AR after 30 days, TAVI has a significantly higher rate of moderate or severe AR then SAVR (4.2% vs 0.4%) [6]. Meanwhile, evidence suggests that even low-grade postoperative AR negatively impacts both functional status and survival after TAVI [5, 7, 8]. AR after TAVI mainly consists of paravalvular leakage, resulting from incomplete circumferential apposition of the prosthetic stent frame to the surrounding calcified tissue. This TAVI-related paravalvular AR is difficult to evaluate by echocardiography, because of acoustic shadowing artifacts originating from the metallic stent frame and the severe calcifications of the native valve that remains in situ. Quantification of paravalvular AR by echocardiography is further complicated by the typical phenotype of this AR, involving multiple, eccentric, and/or wall hugging jets [4]. An emerging novel angiographic method to quantify AR is video-densitometry [9]. Nevertheless, this method is angiography based and thus cannot be used at follow-up.

Another promising tool for assessing AR after TAVI more accurately is cardiac magnetic resonance (MR) imaging [10, 11]. Whereas echocardiography mainly relies on qualitative or semi-quantitative measures [4, 12], and may underestimate or overestimate AR [13–16], cardiac MR provides highly accurate quantitative data on regurgitation severity (i.e., regurgitant fraction and volume) with excellent inter-acquisition repeatability [17] and interobserver and intraobserver agreement [18]. However, AR quantification by cardiac MR has only been validated by in vivo and in vitro studies pertaining to AR in a native valve scenario [18]. Reporting on post-TAVI AR evaluated by cardiac MR might therefore be rather speculative, since there is no data on its accuracy in the presence of transcatheter heart valves (THVs) [10, 11, 13–16, 19–21]. For instance, the metallic stent frame (the backbone of the majority of THVs) could disturb the homogeneity of the magnetic field, which leads to attenuation of the amplitude and errors in the phase of the MR signal. These errors in the phase result in errors of the cardiac MR velocity mapping signal. Moreover, signal void artifacts caused by the stent frame force the imaging slice for through-plane velocity mapping to be placed further away from the zone of the prosthesis-annular apposition, i.e., the location where paravalvular leaks occur, which may lead to underestimation of regurgitation severity [22]. An alternative approach for AR quantification which is less sensitive to stent frame artifacts is calculating the difference between left ventricular and right ventricular stroke volumes based on cine MR imaging. Nevertheless, this is not appropriate in most TAVI patients because of coexisting regurgitation of other heart valves.

Currently, convincing data regarding the most appropriate slice position of the cardiac MR velocity mapping imaging in the presence of a THV are lacking. Various positions have been used, varying between the lower border of the stent frame to the level of the ascending aorta [10, 13–16, 19–21, 23, 24]. Accuracy of aortic regurgitation quantification by MR imaging in the presence of a transcatheter heart valve remains to be established.

The purpose of this pilot study was to evaluate the accuracy of cardiac MR velocity mapping for the quantification of post-TAVI AR in both balloon-expandable and self-expandable valves and to determine at which anatomic level this analysis is most accurate. To this end, cardiac MR velocity mapping of both antegrade and retrograde flow was used in a porcine cadaver heart model in the presence of different types of prosthetic THVs.

## Materials and Methods

### Mock Circulation Setup

**Fig. 1:**
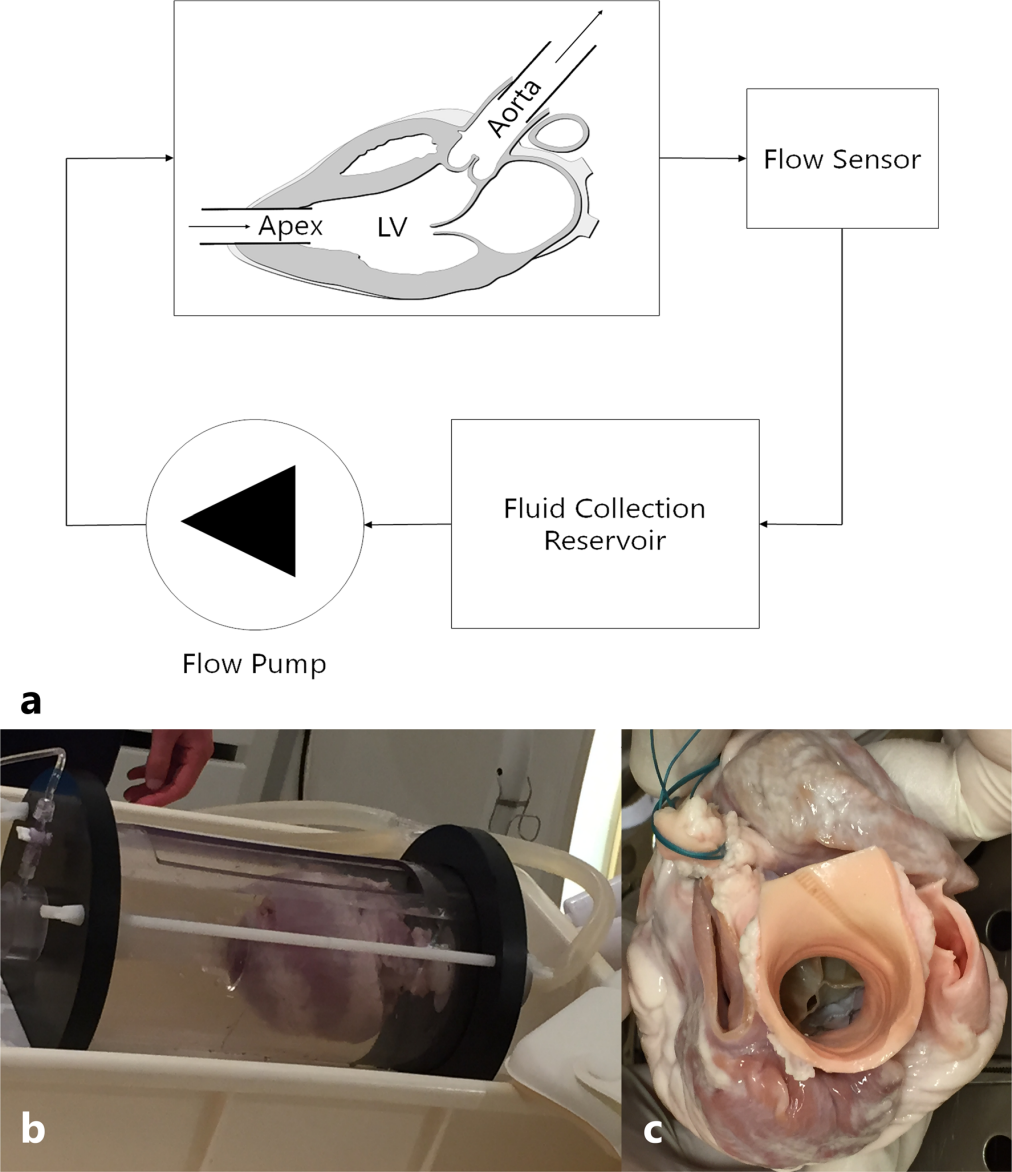
Experimental setup. **a** Schematic representation of the mock circulation loop for continuous flow measurements. **b** Image of the experimental setup. **c** Image of an implanted prosthetic valve

For the experiments, a simple mock circulation loop was used (Fig. 1a). The setup comprised a continuous flow pump, two overflow reservoirs, a fluid collection reservoir, and a testing module connected in series by silicone tubing (internal diameter of 12 mm). A porcine cadaver heart was connected to the outlet of an overflow reservoir at the apex, and the inlet of a second overflow reservoir was connected at the aortic root, approximately 4 cm from the aortic valve (Fig. 1b, c). The overflow reservoirs were used to meet leakage at the tube seams. The mitral valve was sewn up to ensure mitral valve competence.

The system was primed with water. Steady flow was generated by a flow pump. Paravalvular aortic regurgitation was created by an incomplete circumferential apposition between the aortic annulus and THV frame in combination with retrograde flow. Flow rates were measured in real-time by a clamp-on ultrasonic flow sensor (BioProTT™, em-tec GmbH, Finning, Germany). The manufacturer calibrated accuracy had a ± 3% of reading ± 20 ml/min, with a maximum flow rate of 20 l/min. These flow sensor measurements were used as the standard of reference to which the cardiac MR measurements were compared.

### Transcatheter Heart Valves

AR quantification by cardiac MR velocity mapping was performed for two commercially available THVs: the Edwards SAPIEN XT (Edwards Lifesciences, Irvine, CA, USA) and the third-generation Medtronic CoreValve (Medtronic Inc., Minneapolis, MN, USA). Each THV was implanted in a porcine heart by a cardiologist with over 5 years of experience in placing THVs. The SAPIEN XT THV is a balloon-expandable valve that consists of a cobalt chromium frame. The self-expandable CoreValve is composed of a nitinol frame.

### Magnetic Resonance Image Acquisition

All cardiac MR images were acquired on a 1.5 Tesla scanner (Ingenia, Software release 5.1.3, Philips Healthcare, Best, The Netherlands). A gradient echo scout image (slice thickness (ST) 8 mm, field of view (FOV) 311 mm, repetition time (TR) 3 ms, echo time (TE) 1 ms, matrix size 100 × 100, flip angle 60°) was obtained to identify the testing module and guide plane selection for velocity mapping. Flow measurements were acquired by through-plane velocity-encoded phase-contrast imaging (ST 8 mm; FOV 320 mm; TR 5 ms; TE 3 ms; flip angle 12°; matrix size 122 × 122; voxel size 1.25/1.25/8.00 mm). The velocity encoding value (VENC) was set to 80 cm/s. Imaging acquisition was done using the same settings as for a patient with a heart rate of 75/min, with 20 time frames per R-R interval.

Through-plane image selection was performed using both the LV outflow tract and LV inflow/outflow views to ensure perpendicularity to the direction of flow.

### Ex Vivo Measurements

**Fig. 2:**
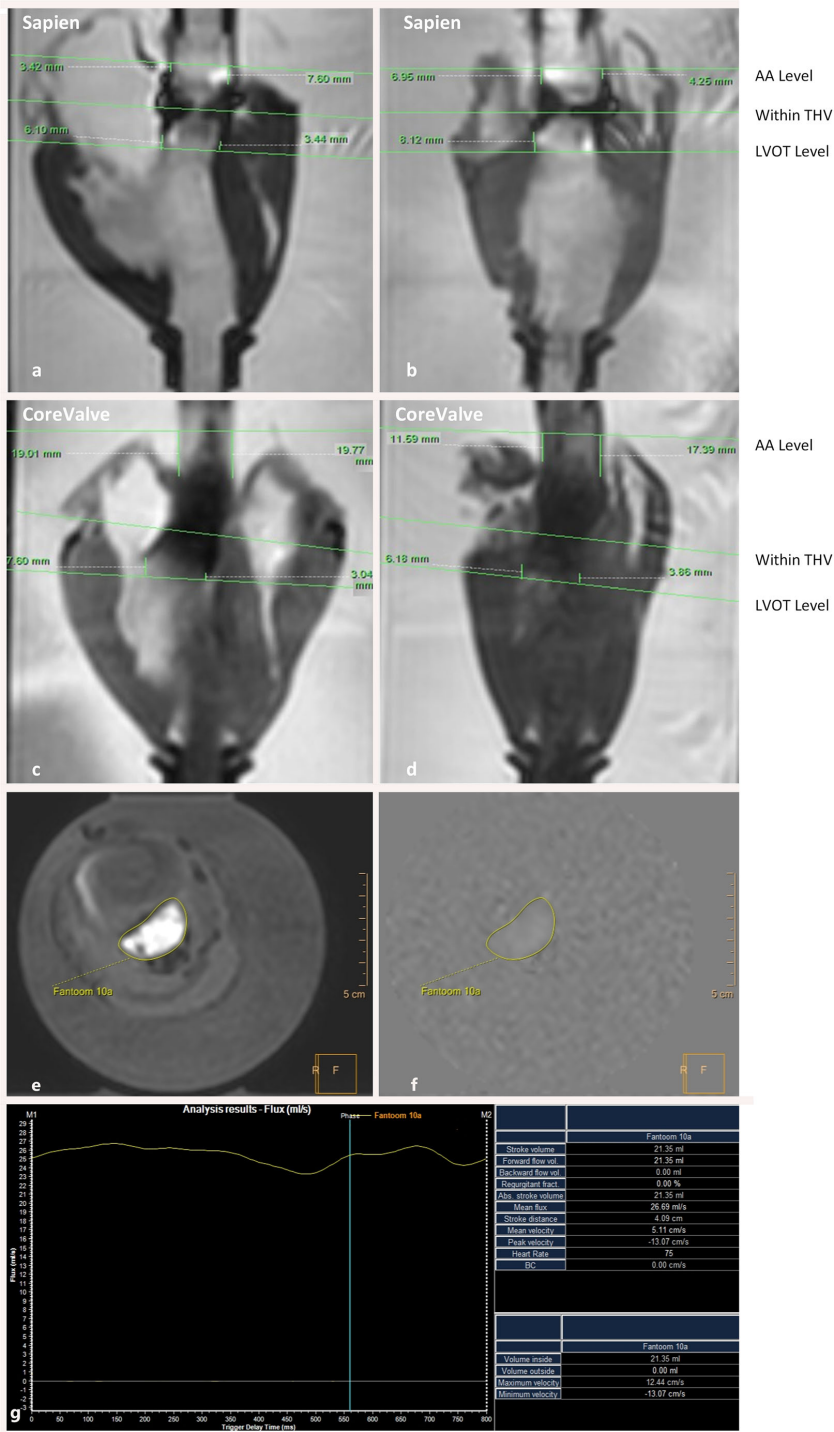
Example of flow analysis by cardiac magnetic resonance. For each THV valve, the 2 perpendicular views with plane selection per level and distance to the THV frame are shown (**a**–**d**). **e** Cardiac MR image of the heart at LVOT level. Yellow circle: ROI. **f** MR velocity–encoded image. Yellow circle: ROI. **g** Curve of flow during one acquisition. AA, ascending aorta; LVOT, left ventricular outflow tract; ROI, region of interest

After placing the mock loop in the MR scanner, the desired flow rate was set. A range of flow rates between 16.67 and 90 ml/s was tested for antegrade flow (AF), and between 16.67 and 50 ml/s for retrograde flow (RF). For clarity, we only reported ranges up to 50 ml/s for AF. The flow ranges were selected according to the AR grading of mild to moderate-severe AR that is used in clinic [4]. Prior to implantation of the THVs, a set of flow measurements was acquired over the native valve with AF. Subsequently, the THVs were implanted and imaged initially with AF, and secondly with RF, thus simulating paravalvular AR. Cardiac MR velocity mapping was performed at 3 different locations: just above the THV (Ascending aorta-side, 3–7.6 mm S3, 11.6–19.8 mm CV), in the THV stent, and just under the THV (left ventricular outflow tract (LVOT)-side, Fig. 2a–d). The exact slice position is shown at Fig. 2a–d. Each acquisition was performed in duplicate. Quantitative analysis of the flow volume was performed by the same experienced radiology technician using the Philips Intellispace Portal Software, version 7.0 (Philips Health Care, Amsterdam, Netherlands) (Fig. 2e, f).

### Statistical Analysis

Data was analyzed with IBM SPSS Statistics software version 21 (IBM Corp., Armonk, NY). Continuous variables are presented as mean ± standard deviation (SD) or median [interquartile range], when considered appropriate. Categorical data are presented as frequency and percentage. The obtained cardiac MR flow measurement volumes were extrapolated to milliliter (ml)/second (s). The accuracy of the measurements at each through-plane level was evaluated by calculating the mean difference ± SD, and the difference in percentage (using the formula: ([reference − measured flow]/reference) × 100%).

Agreement between measurements was evaluated by determining the Pearson correlation coefficient (PCC) of the mean of the paired values of acquisitions 1 and 2 with the reference, simple linear regression, and Bland-Altman analysis. Limits of agreement were determined as mean difference ± 1.96 × SD. The 95% confidence intervals (95% CI) describe the precision, the smaller the interval the higher the precision.

When the slope of the linear regression line differs significantly from 1.0, bias is present. When this bias (mean percentage bias) is proportional with measurements (i.e., of flow) and repeated measurements (high PCC values and low repeatability errors), the bias is caused by within-procedure precision errors, and can be interpreted as a systematic bias and can therefore be used as a correction factor [25].

Repeatability was evaluated by using one-way random intraclass correlation coefficient (ICC), simple linear regression, and construction of Bland-Altman with the average of both acquisitions on the horizontal axis and limits of agreement as mentioned above.

An absolute flow error of ≥ 5 ml/s or error in percentage of > 15% was considered to be clinically significant. Statistical significance was determined at the 95% confidence level (*p* value < 0.05). To account for the small number of samples, nonparametric methods like Wilcoxon were used for comparison.

## Results

In total, 130 flow volumes were measured with cardiac MR; five different continuous flow velocities, for three valves, two flow directions, at three slice levels, and all in duplicate. For each valve, one porcine heart was used.

### Accuracy of Flow Measurements with Cardiac MR

**Fig. 3:**
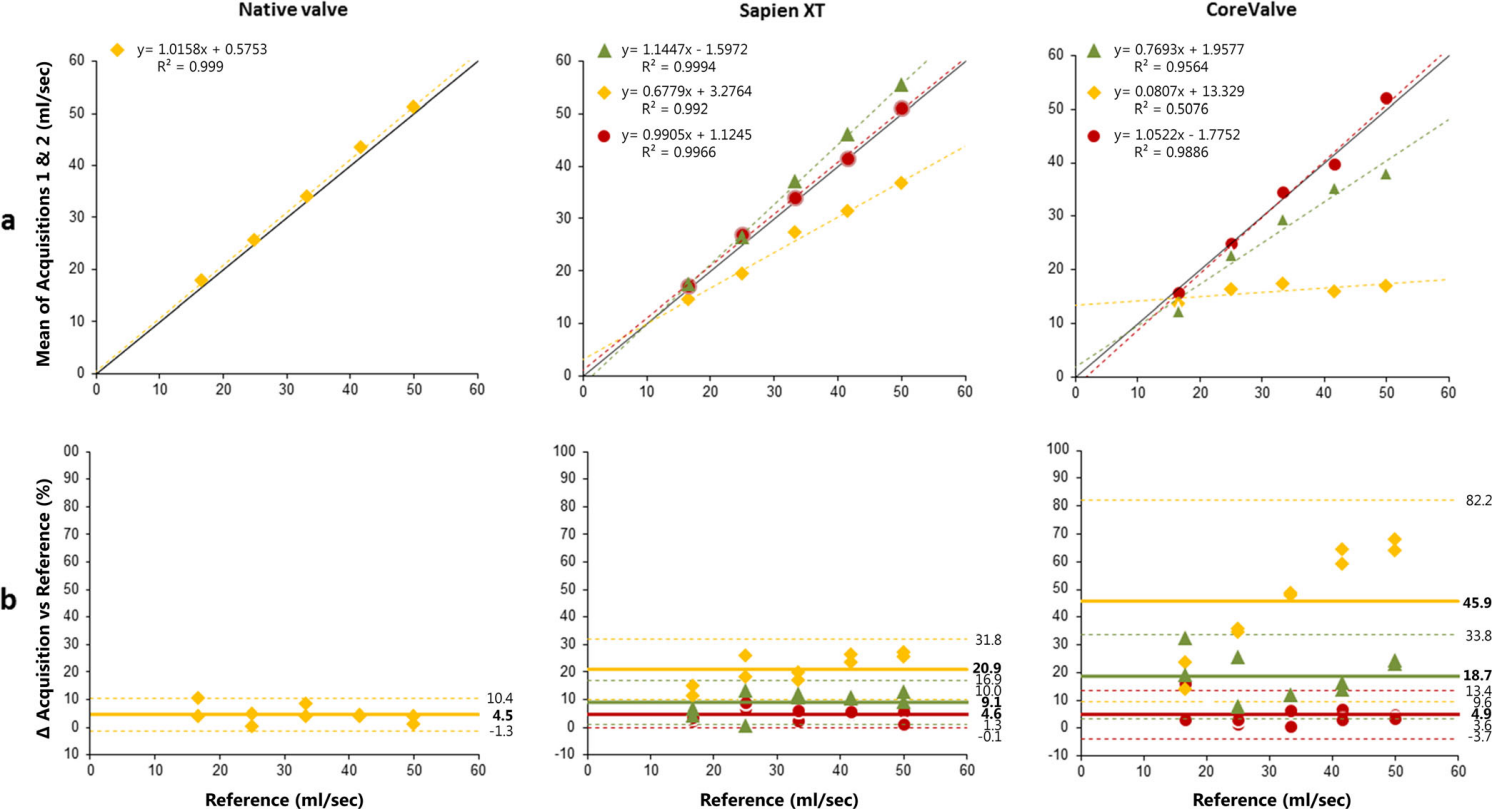
Antegrade flow: Accuracy of flow measurements per level and per valve  = ascending aorta (AA) level  = within THV  = left ventricular outflow tract (LVOT) level. **a** Scatterplot with linear regression line and equation, and R^2^. Unbroken line: reference line of reference flow values; dotted line: regression line. **b** Bland-Altman plot. Unbroken line: mean difference (%), top and bottom dotted lines: limits of agreement (%)

**Fig. 4:**
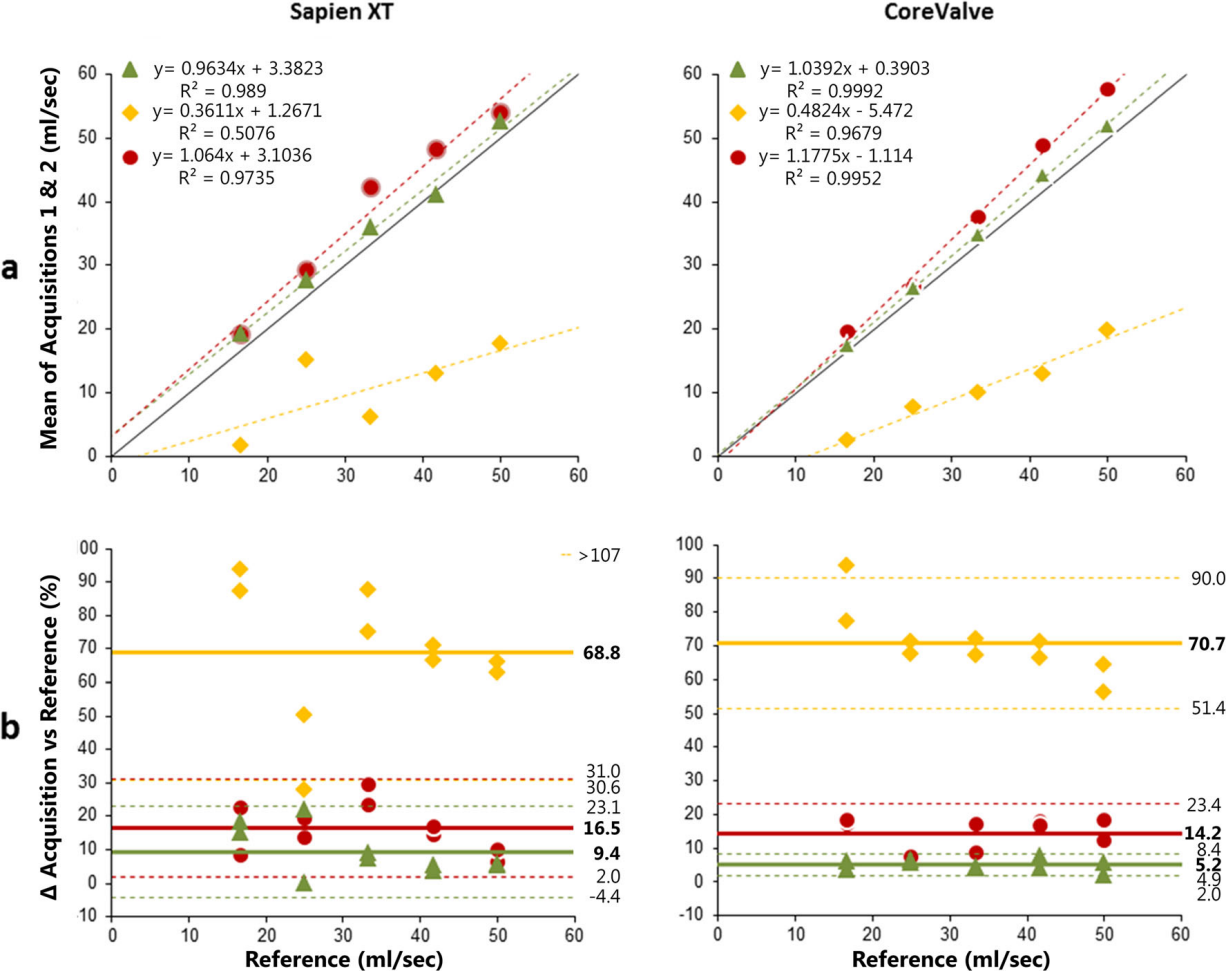
Retrograde flow: Accuracy of flow measurements per level and per valve. Legend is similar to Fig. 3

Accuracy and precision of the flow measurements in both the native and THVs in comparison to the reference flow are presented in Table 1, Figs. 3, and 4. As expected, the acquisition made at the level of the THV frame could not be considered reliable with inharmonious flow curves and mean error percentages of 20.9% to 70.7%, due to severe artifacts.

**Table 1:** Cardiac magnetic resonance accuracy: Acquisition vs reference flow

Valve type	Absolute Δ Acquisitions vs reference (%) ± SD	*p* value of acquisitions vs reference	PCC *r* (3)=	*p* value of PCC	*p* value of Δ AA level vs LVOT level
**Antegrade flow**					
Native valve					
Within valve	4.5 ± 3.0	0.028	0.999	< 0.001	
Sapien XT					0.022
AA level	9.1 ± 4.0	0.007	1.000	< 0.001	
Within THV	20.9 ± 5.5	0.005	0.996	< 0.001	
*LVOT level*	*4.6 ± 2.4*	*0.203*	*0.996*	*< 0.001*	
CoreValve					0.009
AA Level	18.7 ± 7.7	0.007	0.978	0.004	
Within THV	45.9 ± 18.5	0.005	0.713	0.176	
*LVOT level*	*4.9 ± 4.4*	*0.959*	*0.994*	*0.001*	
**Retrograde flow**					
Sapien XT					0.037
*AA level*	*9.4 ± 7.0*	*0.017*	*0.994*	*< 0.001*	
Within THV	68.8 ± 19.5	0.005	0.712	0.177	
LVOT level	16.5 ± 7.4	0.005	0.987	0.002	
CoreValve					0.005
*AA level*	*5.2 ± 1.6*	*0.005*	*1.000*	*< 0.001*	
Within THV	70.7 ± 9.8	0.005	0.984	0.002	
LVOT level	14.2 ± 4.7	0.005	0.998	< 0.001	

*PCC* Pearson correlation coefficient, *AA* ascending aorta, *LVOT* left ventricular outflow tract, *THV* transcatheter heart valve. Δ= difference. Italicized levels and values indicate best slice position

Results for the native valve showed a high correlation (PCC *r* (3) = 0.999, *p* < 0.001), indicating a high degree of consistency between the measured and reference values (Table 1). In Fig. 3, the first column shows the scatter plot and Bland-Altman plot of the cardiac MR measurements in the native valve model for the different flow velocities in comparison to the reference. These plots show a small systematic underestimation and a mean error in percentage of 4.5 with a 95% confidence interval (CI) between − 1.3 and 10.4% (Fig. 3b).

For Sapien XT, AF was best estimated at LVOT level, with no significant differences compared to the reference (*p* = 0.203). As can be deducted from the scatter plot in the middle column in Fig. 3 a, there was a strong correlation between the mean of the two acquisitions and the reference for this slice level (*r* (3) > 0.99). Similarly, the mean error in percentage was significantly lower at LVOT level in comparison to AA level (4.6% ± 2.4% vs 9.1% ± 4.0%, *p* = 0.022, middle column of Fig. 3b), with a smaller 95% CI width (4.7% vs 7.8% for LVOT and AA, respectively). RF results differed only slightly between AA and LVOT, as shown in Table 1 and Fig. 4a. Precision was comparable at both levels as reflected by similar confidence interval width. Only the correlation with the reference values and mean percentage error were slightly higher at the AA level (Table 1 and Fig. 4a, b).

For CoreValve, AF estimation was best at LVOT level as well, with no significant differences compared to the reference (*p* = 0.959). At this LVOT level, cardiac MR measurements showed the highest correlation with reference flow (Table 1, Fig. 3a) and the highest accuracy and precision (Fig. 3b). The RF results were most reliable at the AA level (Table 1, Fig. 4), with the highest precision and accuracy compared to the LVOT level (95%CI 2.0%–8.4% vs 4.9%–23.4%, Fig. 4b).

### Intra-test Repeatability of Cardiac MR Flow Measurements

Results for the intra-test repeatability of the flow measurements of the native and THVs are presented in Table 2. Overall, there was no correlation between the flow velocity and intra-test error, since the scatter plot showed a heteroscedastic scatter pattern.

**Table 2:** Cardiac magnetic resonance intratest repeatability: acquisition 1 vs acquisition 2

Valve type	Absolute Δ (ml/s) ± SD	*p* value of *Δ*	ICC (95% CI)	*p* value of ICC	*p* value of absolute Δ AA level vs LVOT level
**Antegrade flow**					
Native valve					
Within valve	1.6 ± 1.5	0.893	0.988 (0.914–0.999)	< .001	
Sapien XT					0.500
AA level	1.2 ± 1.4	0.225	0.993 (0.951–0.999)	< .001	
Within THV	1.1 ± 0.5	0.686	0.991 (0.936–0.999)	< .001	
LVOT level	2.2 ± 1.8	0.686	0.979 (0.853–0.998)	< .001	
CoreValve					0.500
AA level	2.5 ± 3.4	0.225	0.930 (0.579–0.992)	0.001	
Within THV	1.2 ± 0.9	0.500	0.604 (− 0.292–0.949)	0.079	
LVOT level	1.7 ± 1.0	0.225	0.991 (0.934–0.999)	< .001	
**Retrograde flow**					
Sapien XT					0.686
AA level	2.2 ± 2.4	0.225	0.971 (0.806–0.997)	< .001	
Within THV	2.9 ± 1.9	0.345	0.881(0.364–0.987)	0.005	
LVOT level	1.7 ± 0.5	0.686	0.992 (0.943–0.999)	< .001	
CoreValve					0.686
AA level	0.8 ± 0.8	0.345	0.997 (0.978–1.000)	< .001	
Within THV	2.3 ± 1.2	0.686	0.927 (0.564–0.992)	0.001	
LVOT level	1.3 ± 1.5	0.225	0.993 (0.949–0.999)	< .001	

*ICC* intraclass correlation coefficient, *AA* ascending aorta, *LVOT* left ventricular outflow tract, *THV* transcatheter heart valve

For the native valve, in the AF measurements, the scattering of the differences between the two series is small, indicating high repeatability.

For the Sapien valve, the AF mean absolute intra-test differences were small and differed slightly per slice level (Table 2). The repeatability was best at AA level, with the highest accuracy and precision, and the highest ICC (0.993; *p* < .001). RF measurement repeatability was best at LVOT, with an ICC of 0.992, *p* < .001.

For the CoreValve, the AF flow intra-test differences were lowest at LVOT, with a high precision (95% CI − 4.5 to 2.7 ml/s), and a high correlation (ICC = 0.99). For RF, intra-test results were best at AA.

Overall, there was a high repeatability of cardiac MR measurements at AA and LVOT for all valves and in both flow directions, with all ICCs > 0.93 and absolute differences that were not significant between the AA and LVOT level (Table 2). In addition, the intra-test differences were small.

## Discussion

In this ex vivo pilot study, we investigated the accuracy of cardiac MR velocity mapping for quantification of post-TAVI AR. We report a high level of accuracy and precision between the reference flow and the measured flow by cardiac MR in both THVs and in the native valve, provided that the best slice position is taken into account. This best slice position is similar for both studied THV valves, but is different per flow direction. This is of clinical importance since the ratio between retrograde and antegrade flow defines the regurgitation fraction, resulting in the AR severity, which is related to clinical outcome.

To the best of our knowledge, this is the first study to determine the best slice position for quantification of the retrograde flow by cardiac MR velocity mapping in THVs. In prior studies, AR quantification by cardiac MR has been validated by in vitro and in vivo studies with excellent repeatability and accuracy for native valve AR [18, 26, 27]. However, for post-TAVI AR with a THV in situ, the exact accuracy of cardiac MR flow quantification was hitherto unknown [10, 11, 13–16, 19–21]. The only studies that have been performed to investigate the cardiac MR velocity mapping accuracy for post-TAVI AR have been modality comparison studies. These studies showed that cardiac MR flow measurements correlated better with quantitative flow assessment by angiography than echocardiography [28]. Nevertheless, these correlations and quantifications were not compared to a “gold standard” as reference. Another evidence gap was the lack of studies about the best slice position of cardiac MR velocity mapping (see Table 3).

**Table 3:** Available studies reporting on slice location for quantification of AR with MR flow velocity mapping

Study	Year	*N*	ES/MCV	Slice location for trough-plane velocity mapping	AR ≥ moderate	Cutoff (RF)
Sherif et al. [21]	2011	16	0%/100%	In vicinity of the upper margin of the prosthesis	37.5%	> 30%
Merten et al.** [19]	2013	43	26%/74%	For MCV, just beneath the upper margin of the stent. For ES, corresponding distance from annulus.	18.6%	> 15%
Hartlage et al. [15]	2014	23	NA	2–3 mm above the stent frame	52.0%	> 20%
Orwat et al. [16]	2014	59	100%/0%	10 mm above the above the aortic prosthesis	27.1%	> 20%
Altiok et al.* [14]	2014	71	55%/45%	Just above the cage of the THV	18.0%	> 9%
Ribeiro et al. [13]	2014	42	100%/0%	Sinotubular junction	26.2%	> 20%
Abdel Wahab et al.** [23]	2014	90	62%/38%	Just beneath the upper margin of the stent of the MCV. For ES, corresponding distance from annulus.	1.8% vs 18.2%	> 15%
Crouch et al. [10]	2015	56	100%/0%	4 levels: LVOT, AA, just above and just under THV.	35%	> 20%
Salaun et al. [24]	2015	30	67%/33%	0.5 cm above aortic valve	30%	> 14%
Frick et al.* [20]	2016	69	55%/45%	Just above the cage of the TAVI prosthesis	19.0%	> 9%

*ES* Edwards, *MCV* Medtronic CoreValve, *NA* not available, *RF* regurgitation fraction, *THV* transcatheter heart valve, *LVOT* left ventricular outflow tract, *AA* ascending aorta. A single and double asterisk indicate duplicate reporting. Cutoff (RF) is defined as cut-off value for moderate AR

This pilot study shows that in a systematic and fully controlled laboratory ex vivo setting in a porcine model accurate quantitative estimations of antegrade and retrograde flow across THVs can be achieved by cardiac MR. Furthermore, when AR is measured at the best slice position, the accuracy of the measurement is not significantly affected by susceptibility artifacts due to the metallic stent frame, which might occur < 7 mm of the CoreValve frame and < 30 mm of the Sapien XT frame in a 3.0 Tesla MRI system according to the manufacturers [29, 30].

The measurements in this study were performed in a flow range between 16.7 and 50.0 ml/s, although some measurements, despite measured at the best slice position, still show a clinically significant difference compared to the reference flow with mean absolute percentage biases up to 9.4% (95% CI − 4.4 to 23.1%). This systematic error can be reduced by applying the found linear regression. By correcting the measurements with the valve dependent linear regression correction, the 95% confidence interval shows an error with a maximum of 13.8%, which is a clinically acceptable error percentage. It should be noted that these results are of a proof-of-concept experiment. Consequently, this linear regression correction for the systematic error might be different in real-life practice, due to other MR systems and settings, and the real clinical scenario. For example, our model coronary flow (1.5–3.0 ml/beat in average [31]) and closing volume of the valve (3.3 ± 1.2 ml per beat [32]) were not taken into account. Another possible difference between the used model and clinical scenario might be aortic compliance and aortic root movement that can lead to underestimation of AR up to 20% and 15%, respectively [22, 33]. Nevertheless, by measuring close to the aortic valve and by using a method to compensate for motion of the valve annulus, e.g., a moving slice velocity mapping technique [34], these possible underestimations of AR may be neither present in our model nor in clinical practice.

In our study, the accuracy of flow measurements over the native valve shows a mean systematic error of 4.5 ± 3.0%, which is comparable to a previous study of Søndergaard et al. who reported an error of 5–10% [26]. In another study, a lower average error of only 2% was reported [21]. Nevertheless, those study measurements were performed in a model with only an ascending aorta and a stentless porcine aortic valve and therefore might not have taken into account contributing errors that might be present in a complete native heart as in the current study used.

An important finding of the present study is that the best slice position for AR quantification by cardiac MR velocity mapping differs per flow direction, but is similar for both THV types. For Sapien XT and CoreValve, the best slice position for antegrade flow is at LVOT level, and for retrograde flow at AA level. At these levels, high accuracy and precision, and excellent repeatability, are achieved with errors less than 5 ml/s and therefore considered not to be clinically significant. The found difference in best slice position depending on flow direction might be explained by the fact that the prosthetic valve is introducing artifacts and measuring errors. When retrograde flow is measured at AA level, the flow has not passed the valve yet. Similarly, the antegrade flow at LVOT level is measured before the blood flow is passing the valve. Therefore, laminar flow and not the error-prone non-laminar flow is measured. Non-laminar flow may lower the accuracy due to local eddies which do not pass perpendicularly through the plane of acquisition. Another new technique to tackle this non-laminar flow problem is 4D flow. 4D flow accounts for differences in flow patterns and may therefore be more accurate for non-laminar flow [35, 36]. A recent systematic review of Crandon et al. described that 4D flow MR is reliable for flow quantification and regurgitation across the aortic valve [37]. However, to the best of our knowledge, 4D flow MR has not yet been validated in patients with a prosthetic valve. Thus, possible artifacts and distortions due to the prosthetic valve are unknown. Another promising method to quantify AR is video-densitometry [9]. A major advantage of this technique is that it is angiography based, and therefore available in all procedures. Conversely, this also means it cannot be used at follow-up.

The results of this study for evaluating the accuracy of AR could be of clinical importance, particularly the found difference in best slice position depending on flow direction. The rapidly increasing numbers of TAVI procedures will see the greater need for accurate post-procedure imaging of paravalvular AR. Confirmation of our results in future clinical studies would be of clinical importance; since then, AR severity can be assessed more accurately by measuring the flow both at the AA level and at the LVOT level.

The current study has several important limitations. First, we used a porcine heart as model to mimic the human heart as closely as possible. Although the human heart is very similar, there are some interspecies differences, like a shorter ascending aorta and smaller sinotubular junction [38]. However, this only influences the difficulty of the THV implantation, not the flow measurements. Secondly, the Sapien S3 and Evolut Pro are currently the mostly used valves. Nonetheless, despite some small differences in the stent frame between the older and newer valves, the manufacturers of the newer valves claim susceptibility artifacts by the metallic frame to be similar. Other differences between the older and the newer valves, such as addition of a sealing skirt, might create flow disturbances and thereby potentially affect the measurements by cardiac MR velocity mapping. However, the skirt itself is less likely to affect the flow measurement, since this skirt is not a moving element and is not located directly in the slice plane. Thirdly, this model is not able to reflect all possible errors that might affect the AF and RF measurements in patients, such as cardiac and respiratory motion, laminar blood flow (due to lower viscosity of water than whole blood), pulsatile flow, annular calcification, and inaccuracies due to windkessel and coronary flow. Also, given the complexity and heterogeneity of leaflet and annular calcification in aortic valve stenosis in patients, paravalvular AR may take the form of a three-dimensional tract and therefore may be underestimated as well in the used model. Another limitation of this study is that the studied flowrates do not correspond to the full range of flowrates observed in vivo. In vivo, AF flow rates up to and beyond 200 ml/s are commonly reached in the systolic phase in healthy patients, and RF flowrates over 100 ml/s in diastolic phase correspond to severe aortic regurgitation. These higher flowrates introduce different fluid dynamic conditions, and require different MRI data acquisition settings (i.e., increased VENC). This limitation could introduce possible errors such as an increased signal-to-noise ratio when the measurement method of this study is directly translated in clinic. Despite these limitations, our study is an accurate, systematic, and fully controlled laboratory study that can serve as a good reference point for future studies, in which potentially additional errors should be evaluated further. In addition, this study can be used as a start for future studies to finally better understand the relation between post-TAVI AR and its impact on functional status and survival in clinical patients.

In conclusion, cardiac MR velocity mapping enabled accurate, precise, and repeatable quantification of (para)valvular regurgitation in an ex vivo model with two commonly used prosthetic aortic valves, when corrected for a systematic error and the use of the best cardiac MR slice position.

## Abbreviations

AR: Aortic regurgitation
AF: Antegrade flow
RF: Retrograde flow
MR(I): Magnetic resonance (imaging)
THV: Transcatheter heart valve
TAVI: Transcatheter aortic valve implantation
SAVR: Surgical aortic valve replacement
ST: Slice thickness
FOV: Field of view
TR: Repetition time
TE: Echo time
VENC: Velocity encoding value
ICC: Intraclass correlation coefficient
PCC: Pearson correlation coefficient
